# BTN2A2 protein negatively regulates T cells to ameliorate collagen-induced arthritis in mice

**DOI:** 10.1038/s41598-021-98443-5

**Published:** 2021-09-29

**Authors:** Xueping He, Rong Hu, Peng Luo, Jie Gao, Wenjiang Yang, Jiaju Li, Youjiao Huang, Feng Han, Laijun Lai, Min Su

**Affiliations:** 1 Department of Human Histology and Embryology, School of Basic Medical Sciences, Center for Tissue Engineering and Stem Cell Research, Guizhou Medical University (North Campus), Guiyang, 550025 Guizhou China; 2 Translational Medicine Research Center, Guizhou Medical University, Guiyang, 550025 Guizhou China; 3 School of Public Health, The Key Laboratory of Environmental Pollution Monitoring and Disease Control, Ministry of Education/ Guizhou Provincial Engineering Research Center of Food Nutrition and Health Guizhou Medical University, Guiyang, 550025 Guizhou China; 4 State Key Laboratory of Functions and Applications of Medicinal Plants, Guizhou Medical University, Guiyang, 550014 China; 5 Department of Neurosurgery, The Affiliated Hospital of Guizhou Medical University, Guiyang, 550004 Guizhou China; 6 Department of Allied Health Science, University of Connecticut, 1390 Storrs Road, Storrs, CT 06269 USA; 7 Key Laboratory for Adult Stem Cell Translational Research, Chinese Academy of Medical Sciences, Guiyang, 550004 Guizhou China

**Keywords:** Immunology, Rheumatology

## Abstract

Rheumatoid arthritis (RA) is an autoimmune disorder characterized by persistent inflammatory responses in target tissues and organs, resulting in the destruction of joints. Collagen type II (CII)-induced arthritis (CIA) is the most used animal model for human RA. Although BTN2A2 protein has been previously shown to inhibit T cell functions in vitro*,* its effect on autoimmune arthritis has not been reported. In this study, we investigate the ability of a recombinant BTN2A2-IgG2a Fc (BTN2A2-Ig) fusion protein to treat CIA. We show here that administration of BTN2A2-Ig attenuates established CIA, as compared with control Ig protein treatment. This is associated with reduced activation, proliferation and Th1/Th17 cytokine production of T cells in BTN2A2-Ig-treated CIA mice. BTN2A2-Ig also inhibits CII-specific T cell proliferation and Th1/Th17 cytokine production. Although the percentage of effector T cells is decreased in BTN2A2-Ig-treated CIA mice, the proportions of naive T cells and regulatory T cells is increased. Furthermore, BTN2A2-Ig reduces the percentage of proinflammatory M1 macrophages but increases the percentage of anti-inflammatory M2 macrophages in the CIA mice. Our results suggest that BTN2A2-Ig protein has the potential to be used in the treatment of collagen-induced arthritis models.

## Introduction

Autoimmune diseases including rheumatoid arthritis (RA) are characterized by chronic and persistent inflammation in target tissues and organs. During RA progression, the synovial lining layer of the inflamed joints increases its thickness as a result of synovial hyperplasia and inflammation^1^. Collagen-induced arthritis (CIA) is a well-characterized mouse model for human RA, in which injection of type II collagen (CII) into male DBA/1 mice induces swelling and progressive inflammation in joints, leading to arthritis. The CIA model has been used extensively to identify potential pathogenic mechanisms, and to design and test new therapeutics for human RA^2,3^.

Studies have shown that T cells play a pivotal role in the pathogenesis of RA^4–7^. T cells are regulated by costimulatory and coinhibitory molecules. T cell coinhibitory molecules are critical for maintaining peripheral tolerance to avoid autoimmune disease. Among the T cell regulators, the B7 family members are of central importance. The importance of the B7 family is illustrated by the FDA's approval of several drugs for the treatment of immune-related diseases including RA by targeting the ligands or receptors of the B7 family members, such as CTLA-4.

Butyrophilins constitute a family of transmembrane proteins consisting of butyrophilin (BTN), BTN-like (BTNL), and selection and upkeep of intraepithelial T cell (SKI NT) proteins^8–14^. The extracellular BTN domains share sequence and structural similarity with the B7 family members. The functions of some BTN and BTNL members also like the B7 family members, either inhibiting or stimulating T cell activation and proliferation^15–25^. Therefore, BTN and BTNL molecules have been proposed to belong to an extended B7 family^23,26,27^.

It has been reported that BTN2A2 is expressed on B cells, DCs, and macrophages and the expression levels are upregulated upon activation^19,21^. BTN2A2 is also expressed on thymic epithelial cells (TECs)^21^. The BTN2A2 putative receptor is expressed on activated T cells^21^. BTN2A2 protein inhibits T cell proliferation, metabolism and Th1 cytokine production^21^. BTN2A2 inhibits TCR signaling molecules including CD3s, Zap70, and subsequent Erk1/2 activation^19^. BTN2A2 also reduces PI3K and Akt signaling^19^. BTN2A2 induces the production of CD4^+^CD25^+^Foxp3^+^ regulatory T cells (Tregs)^19^. *Btn2a2*-deficient mice have enhanced effector CD4^+^ and CD8^+^ T cell responses, impaired CD4^+^ regulatory T cell induction, and exacerbated experimental autoimmune encephalomyelitis^8^.

However, the ability of BTN2A2 protein to affect immune responses in vi-vo has not been reported. It has been reported that *Btn2a2* is a RA-associated gene^28^. In this study, we produced a recombinant mouse BTN2A2-IgG2a Fc (mBTN2A2-Ig) fusion protein and set out to investigate the ability of the protein to affect CIA.

## Results

### BTN2A2 protein is expressed on T cells and APCs

**Figure 1:**
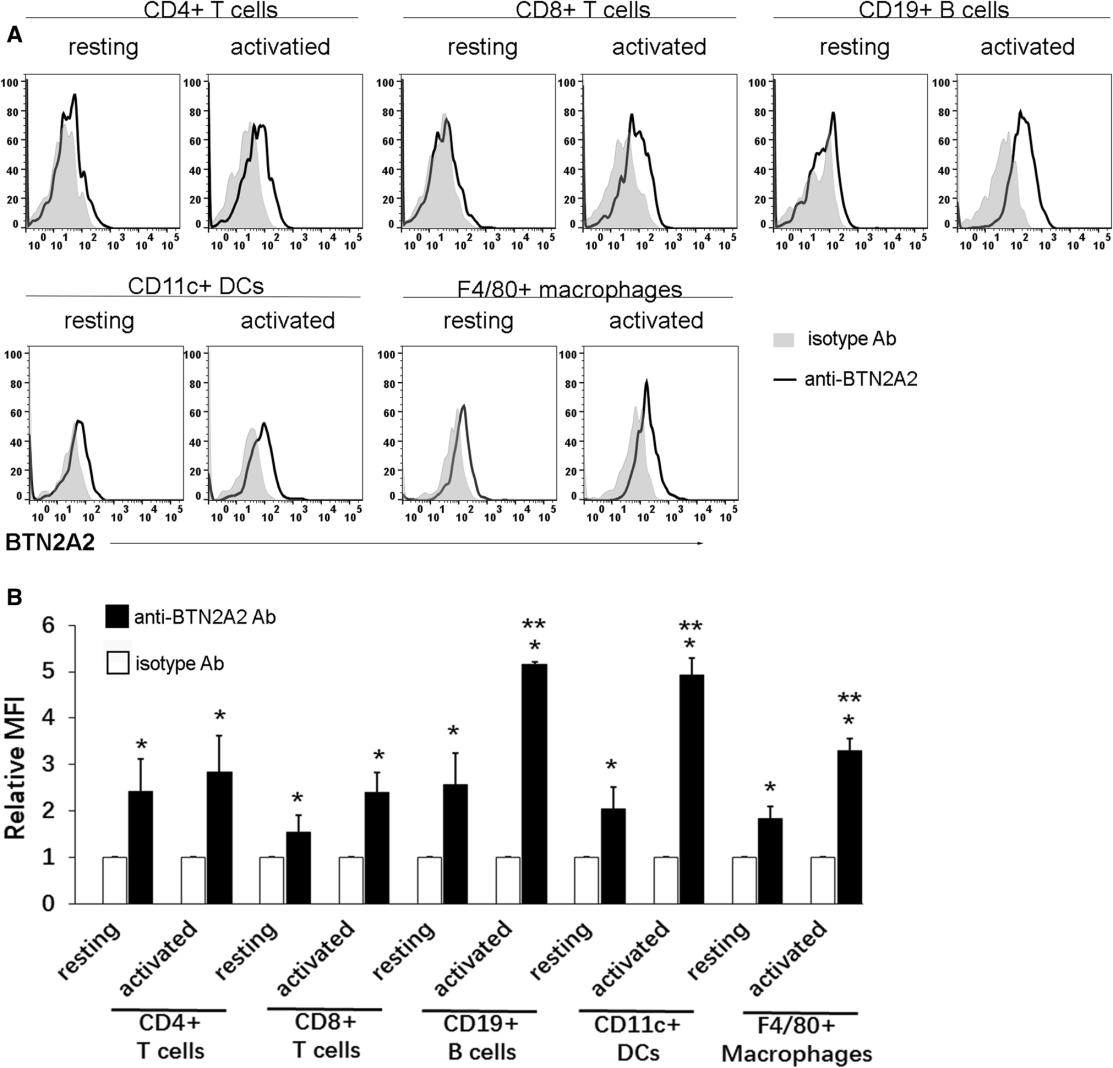
The expression pattern of BTN2A2 protein on immune cells. (**A**) Splenocytes from C57BL/6 mice that were freshly harvested were used for resting immune cells. To activate T cell, splenocytes were incubated with anti-CD3 antibody (1 μg/ml) for 3 days. To activate B cells, DCs, monocytes and macrophages, splenocytes were stimulated with LPS (5 μg/ml) for 3 days. The resting and activated immune cells were stained with anti-BTN2A2 or isotype antibody as well as anti-CD4, CD8, CD19, CD11c or F4/80 antibody to identify immune cells. The cells were analyzed for BTN2A2 protein expression by flow cytometry. Representative flow cytometric profiles, and (**B**) Statistical analysis for the expression levels BTN2A2 on immune cells. Data are presented as relative fluorescence intensity (RFI) for the cells stained with antiBTN2A2 antibody versus cells stained with isotype antibody. *P < 0.05 compared with isotype antibody. **P < 0.05 compared with resting cells. The data are representative of three independent experiments with similar results (n = 3 each experiment).

We first analyzed the expression of BTN2A2 protein on the cell surface of mouse immune cells by flow cytometry using a commercially available anti-mouse BTN2A2 antibody. BTN2A2 protein was expressed weakly on the cell surface of resting CD4^+^ or CD8^+^ T cells and the expression levels were slightly increased upon activation by anti-CD3 antibody although the differences did not reach significant levels (Fig. 1A,B). BTN2A2 protein was also expressed weakly on resting CD11c^+^ dendritic cells (DCs), F4/80^+^ macrophages, and CD19^+^ B cells, and the expression levels on these cells were significantly upregulated upon activation by LPS. (Fig. 1A,B). These results suggest that endogenous BTN2A2 is an integral cell surface protein that is expressed on resting and activated T cells, DCs, macrophages and B cells.

### The putative BTN2A2 receptor is expressed on activated T cells

**Figure 2:**
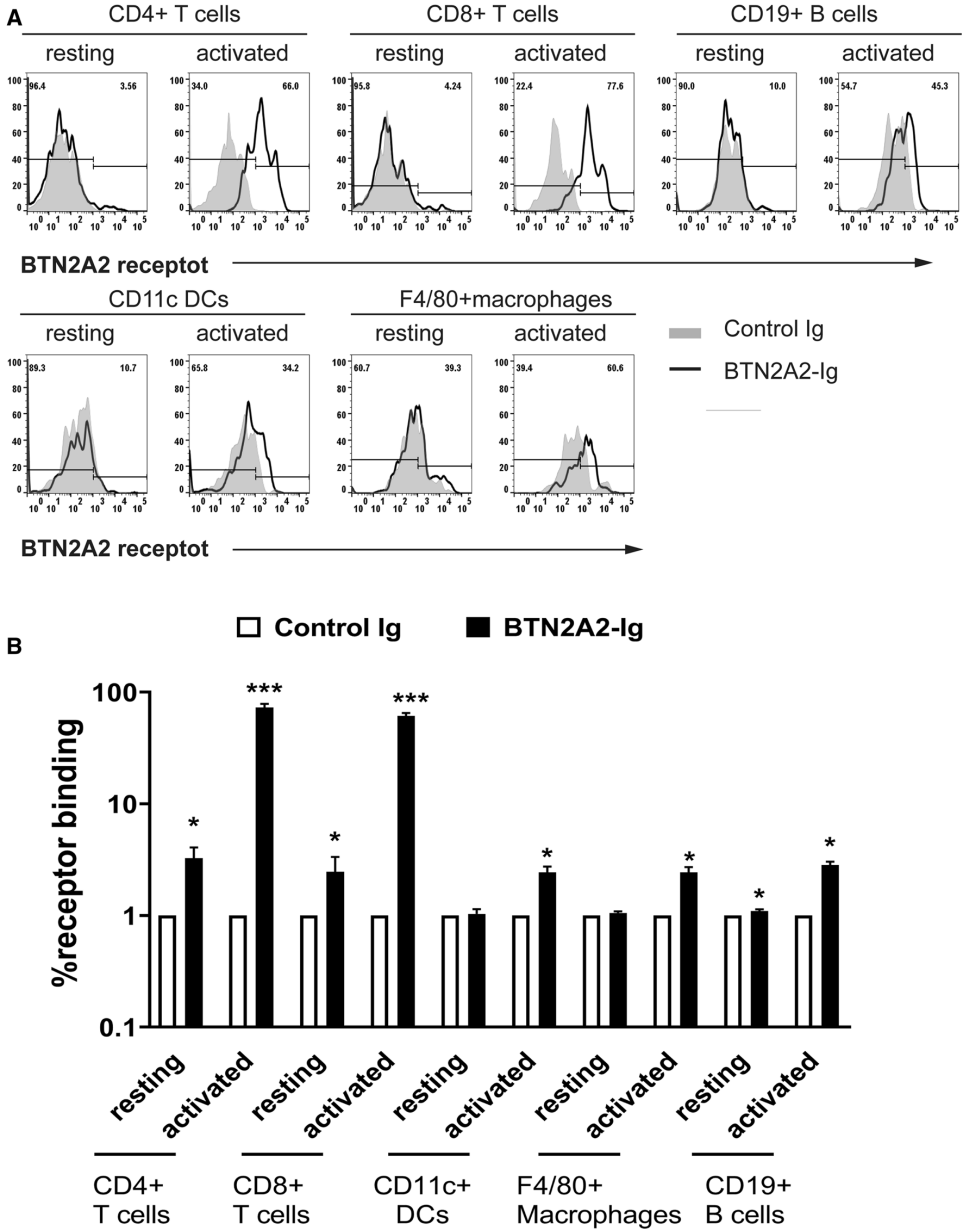
The expression pattern of the putative BTN2A2 receptor on immune cells. Resting and activated immune cells were prepared as Fig. 1. The immune cells were incubated with biotinylated BTN2A2-Ig or control Ig protein for 4 h, followed by PE conjugating streptavidin and antibodies against CD4, CD8, CD11c or F4/80 or CD19. (**A**) Representative flow cytometric profiles and (**B**) statistical analysis showing the binding of BTN2A2-Ig or control Ig to resting and activated immune cells. Data are presented as the percentage of cell binding of BTN2A2-Ig versus control Ig. *P < 0.05 compared with control Ig. **P < 0.05 compared with resting cells. The data are representative of three independent experiments with similar results. (n = 3 each experiment).

To determine the expression pattern of the putative mBTN2A2 receptor, we constructed a mouse BTN2A2-Ig fusion protein that contains the extracellular domain of BTN2A2 and the constant region of mouse IgG2a (Supplementary Fig. 1). Purified BTN2A2-Ig fusion protein and control mouse IgG2a Fc (control Ig) were biotinylated. Splenocytes from C57BL/6 mice were stained with the biotinylated proteins, followed by Streptavidin-PE. The binding of BTN2A2-Ig or control Ig to immune cells was analyzed by flow cytometry. We found that BTN2A2-Ig weakly bound to resting CD4^+^ and CD8^+^ T cells but the binding to anti-CD3 antibody activated CD4^+^ and CD8^+^ T cells was significantly increased (Fig. 2A,B). The results suggest that T cells, especially activated CD4 and CD8 T cells, express the putative BTN2A2 receptor.

We also analyzed the expression of the putative BTN2A2 receptor on other immune cells. We found that BTN2A2-Ig did not bind to resting CD11c^+^ DCs, F4/80^+^ macrophages, and CD19^+^ B cells but bound weekly to these cells upon activation by LPS (Fig. 2A,B). The data suggest that activated DCs, macrophages and B cells express low levels of the putative BTN2A2 receptor. Our data are in agreement with previous reports^19,21^.

### BTN2A2-Ig fusion protein has inhibitory effect on T cells in vitro

**Figure 3:**
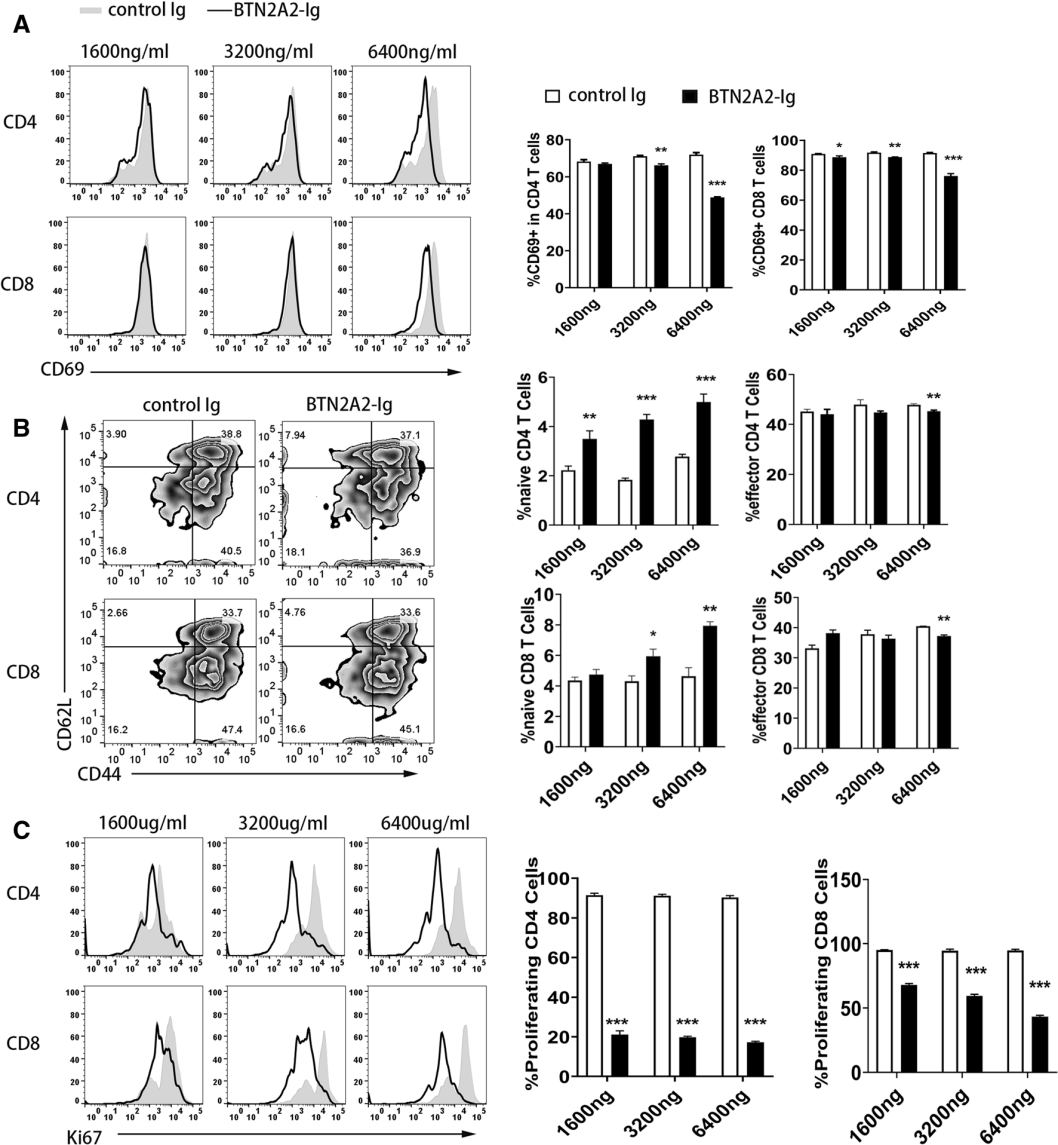
The effects of BTN2A2-Ig protein on murine T cell activation and proliferation in vitro. Splenocytes from C57BL/6 mice were incubated on 96-well plate precoated with 0.5 μg/ml of anti-CD3 antibody and the indicated dose of BTN2A2-Ig or control Ig. The cells were analyzed for (**A**) the expression of CD69 18 h later and (**B**) the percentages of CD44^lo^CD62L^hi^ naïve and CD44^hi^CD62L^lo^ effective memory CD4 and CD8 T cells 72 h later, and (**C**) the expression of Ki67 by CD4 or CD8 positive T cells 72 h later. The data are representative of three independent experiments with similar results. (n = 3 each experiment) *P < 0.05, **P < 0.01 and ***P < 0.001, compared with control Ig.

To determine whether BTN2A2-Ig protein have an affect on T cell activation. The spleen of C57BL/c mice was taken and T cells were stimulated by anti-CD3 antibody and cultured with graded dose of BTN2A2-Ig (1600 ng, 3200 ng and 6400 ng/ml) or control Ig of equal molar mass for 3 days. Since CD69 is an early marker of T cell activation, the expression of CD69 in CD4^+^ T cells and CD8^+^ T cells was analyzed after 16–18 h of culture. As shown in Fig. 3A, the expression of CD69 on CD4^+^ T cells and CD8^+^ T cells significantly decreased in a dose-dependent manner after BTN2A2-Ig treatment. The results showed that BTN2A2 could inhibit the activation of CD4^+^ T cells and CD8^+^ T cells.

In order to further verify the effect of BTN2A2-Ig protein on T cell activation, we analyzed the effect of BTN2A2-Ig on the expression levels of CD44 and CD62L. As shown in Fig. 3B, BTN2A2-Ig treatment increased the percentage of CD44^lo^CD62L^hi^ naive CD4 and CD8 T cells, but decreased the percentage of CD44^hi^CD62L^lo^ effective memory CD4 and CD8 T cells (Fig. 3B). The results further showed that BTN2A2 inhibited the activation of CD4 and CD8 T cells.

To investigate whether BTN2A2-Ig protein affects the proliferation of T cells. Stimulate T cells with 5 µg/ml anti-CD3 antibody and culture splenocytes with graded doses of BTN2A2-Ig or control Ig for 3 days. The cells were stained with Ki67, a proliferating cell marker. The expression of Ki67^+^ cells in CD4^+^ or CD8^+^ T cells was analyzed by flow cytometry. As shown in Fig. 3C, different doses of BTN2A2-Ig protein have inhibitory effects on CD4^+^ and CD8^+^ T cells induced by a-CD3 and are dose-dependent. In conclusion, our data confirmed that BTN2A2 inhibits TCR-mediated activation and proliferation of CD4 and CD8 T cells in vitro.

Then we explored the effects of BTN2A2-Ig protein on Th1 and Th17 cytokine-producing and the differentiation of macrophages in vitro. Spleen cells from C57BL/6 mice were incubated on a 96-well plate pre-coated with 1 μg/ml anti-CD3 antibody and the specified dose of BTN2A2-Ig or control Ig (10 µg/ml). We first examined Th1 or Th17 cytokine-producing. As shown in (Supplementary Fig. 2A–D), BTN2A2-Ig protein reduced Th1 and Th17 cytokine production. Next, we examined whether BTN2A2-Ig affects macrophage polarization in vitro. Our results suggest that BTN2A2-Ig protein reduced the percentage of CD206^lo^MHCII^hi^ M1 macrophages but increased the percentage of CD206^hi^MHCII^lo^ M2 macrophages in vitro (Supplementary Fig. 2E,F).

### Administration of BTN2A2-Ig fusion protein ameliorates CIA in mice

**Figure 4:**
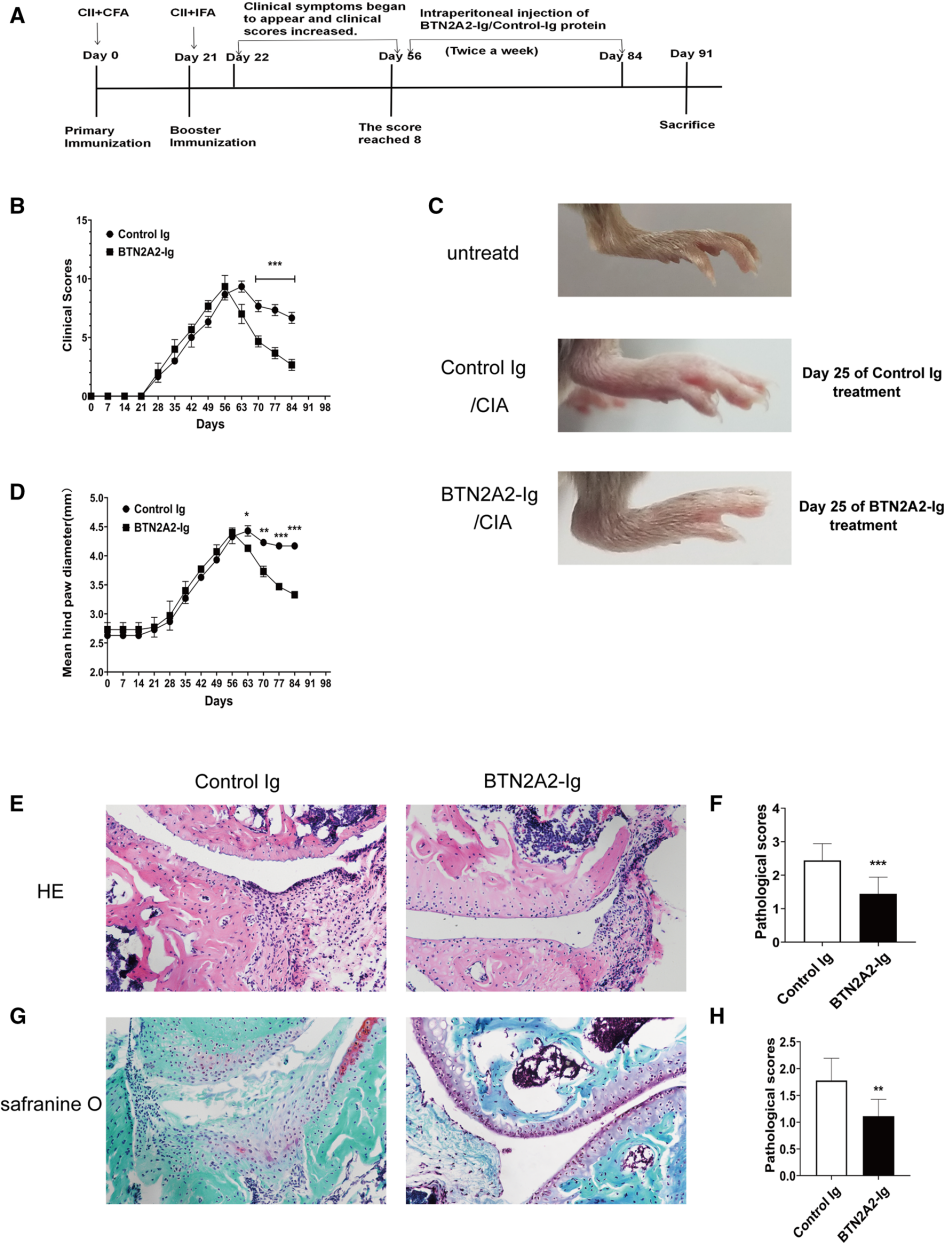
Administration of BTN2A2-Ig alleviates CIA in mice. Male 6–8-week-old DBA/1J mice were immunized with 200 μg of bovine collagen II emulsified in CFA on day 0, and rechallenged with bovine collagen II emulsified in IFA on 21 days. When clinical scores were reached 8, The mice were injected i.p. with 15 μg BTN2A2-Ig or control Ig protein two times per week for 4 weeks. CIA development was monitored over time. (**A**) A flowchart for the generation of CIA model mice, (**B**) CIA clinical scores, (**C**) representative images of hind paws from control Ig- or BTN2A2-Ig protein-treated CIA mice and untreated normal mice, and (**D**) statistical analysis of the thickness of hind paws. (**E**–**H**) The rear paws were harvested from the mice on day 84. (**E**,**F**) H&E staining of (**E**) the sections of metatarsophalangeal joint from control Ig- or BTN2A2-Ig-treated CIA mice, and (**F**) pathological scores are shown. (**G**,**H**) Safranin O-fast green staining of (**G**) representative hind paws, and (**H**) pathological scores are shown. The data are representative of three independent experiments (n = 6 each experiment each group) with similar results.

Having demonstrated that BTN2A2-Ig inhibits T cell activation and proliferation in vitro*,* we examined whether BTN2A2-Ig could treat autoimmune arthritis. Establishment of CIA model (a flowchart for the generation of CIA model mice is provided in Fig. 4A): immunization with bovine type II collagen and CFA on day 0 and booster immunization with bovine type II collagen and IFA on 21st day, after booster immunization the Mouse slowly developed joint erythema and clinical scores started to increase, with peak incidence when the scores reached 8 on 56th day(Fig. 4B), in which the mice were randomly divided and injected intraperitoneally (i.p.) with either control Ig or BTN2A2-Ig protein (15 μg/each mouse) two times per week for 4 weeks. We can see from Fig. 4B that the clinical scores of the mice gradually decreased during the process of treatment from 56th day to 84th day and tended to be moderate. The representative images of the hind paws are shown in Fig. 4C. Untreated normal mice were also used as a control. Statistical analysis shows that BTN2A2-Ig decreased the mean hind paw diameter, as compared to control Ig treatment group (Fig. 4D).

The rear paws were harvested from the mice on day 84 and subjected to histological examination. H&E staining of arthritic paw joints from control Ig-treated CIA mice showed severe infiltration of mononuclear cells and synovial hyperplasia (Fig. 4E). In contrast, BTN2A2-Ig treatment significantly reduced these features (Fig. 4E). Consequently, the pathological scores in BTN2A2-Ig-treated mice was significantly decreased (Fig. 4F). Furthermore, Safranin O-fast green staining revealed that control Ig-treated CIA mice had cartilage destruction as shown in poor pink color, whereas BTN2A2-Ig-treated mice had significantly reduced cartilage destruction (Fig. 4G,H). Taken together, our results suggest that in vivo administration of BTN2A2-Ig protein attenuates established CIA.

### BTN2A2-Ig inhibits T cell activation and proliferation but increases the proportion of Tregs in CIA mice

**Figure 5:**
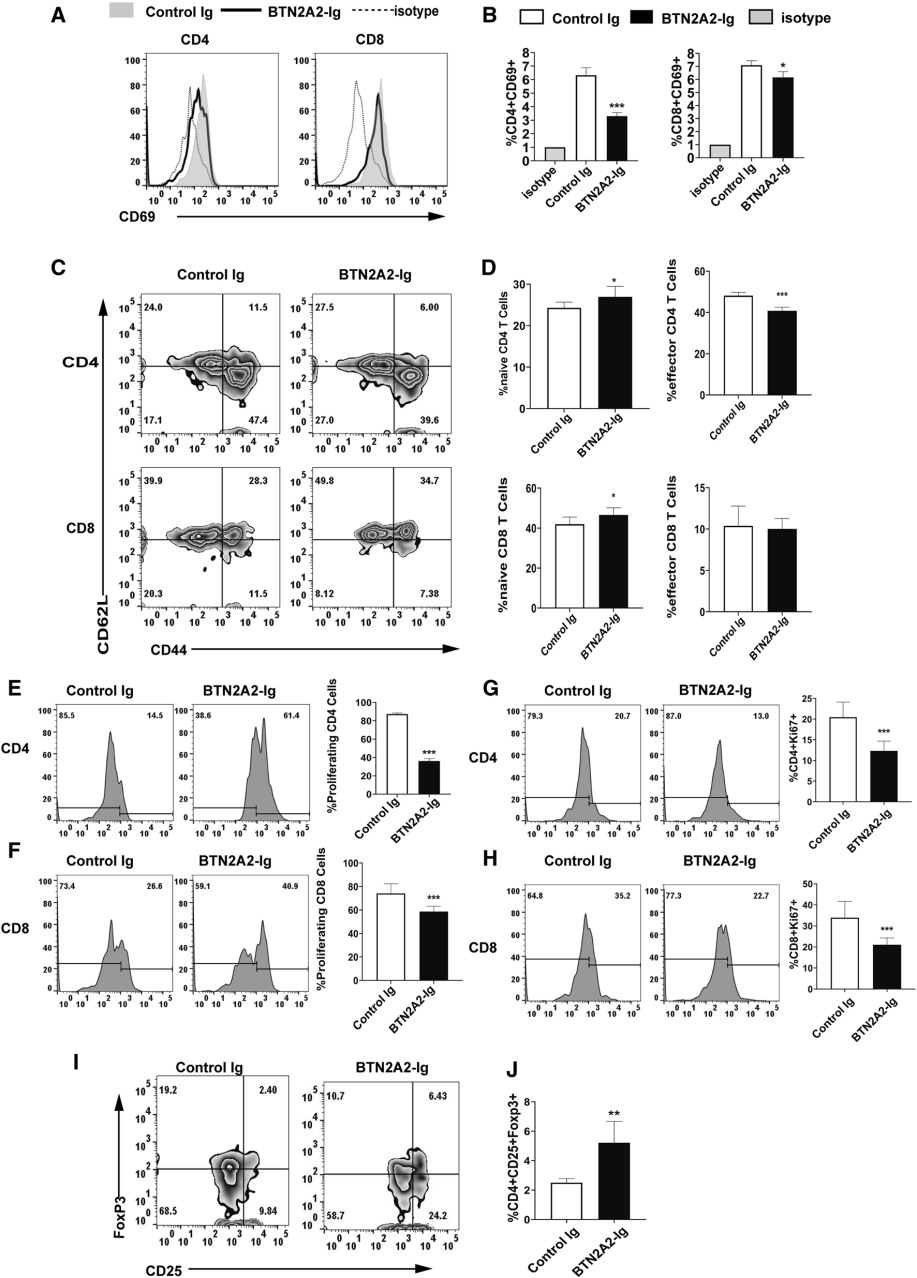
BTN2A2-Ig inhibits T cell activation and proliferation in CIA mice. DBA/1J CIA mice were treated with BTN2A2-Ig or control Ig as in Fig. 4. The spleens were harvested 35 days later. Spleen cells were stained with anti-CD69 or isotype antibodies as well as anti-CD4, CD8 antibody to identify activated cells. The splenocytes were analyzed for (**A**,**B**) the expression of CD69 by CD4 and CD8 T cells, (**C**,**D**) the percentages of CD44^lo^CD62L^hi^ naïve and CD44^hi^CD62L^lo^ effector memory in CD4 and CD8 T cells, and T cell proliferation by (**E**,**F**) CFSE dilution assay and (**G**,**H**) Ki67 staining. (**I**,**J**) The splenocytes were also analyzed for the percentages of CD25^+^FoxP3^+^CD4^+^ Tregs. The data are expressed as the mean ± SD and representative of three independent experiments with similar results. (n = 6 each experiment each group) *P < 0.05, ***P < 0.001 compared with control-Ig.

We then analyzed T cell activation and proliferation in the control Ig and BTN2A2-Ig treated CIA mice. BTN2A2-Ig treatment significantly reduced the expression of CD69 by CD4 and CD8 T cells (Fig. 5A,B), increased the percentages of CD44^lo^CD62L^hi^ naive CD4 and CD8 T cells but decreased the percentages of CD44^hi^CD62L^lo^ effective memory CD4 and CD8 T cells (Fig. 5C,D). BTN2A2-Ig treatment also inhibited CD4 and CD8 T cell proliferation as determined by CFSE dilution assay (Fig. 5E,F) and Ki67 staining (Fig. 5G,H). Our results suggest that BTN2A2-Ig treatment inhibits the activation and proliferation of T cells in vivo, consistent with our in vitro data (Fig. 3),

Since Tregs play a key role in immune tolerance induction and CD4^+^CD25^+^FoxP3^+^ cells are the most profoundly characterized Tregs^29^, we then evaluated CD4^+^CD25^+^FoxP3^+^ Tregs. We found that BTN2A2-Ig treatment increased the percentage of Tregs in the spleen (Fig. 5I,J).

### BTN2A2-Ig reduces Th1 and Th17 cytokine production in CIA mice and inhibits CII-specific T cell proliferation and cytokine production

**Figure 6:**
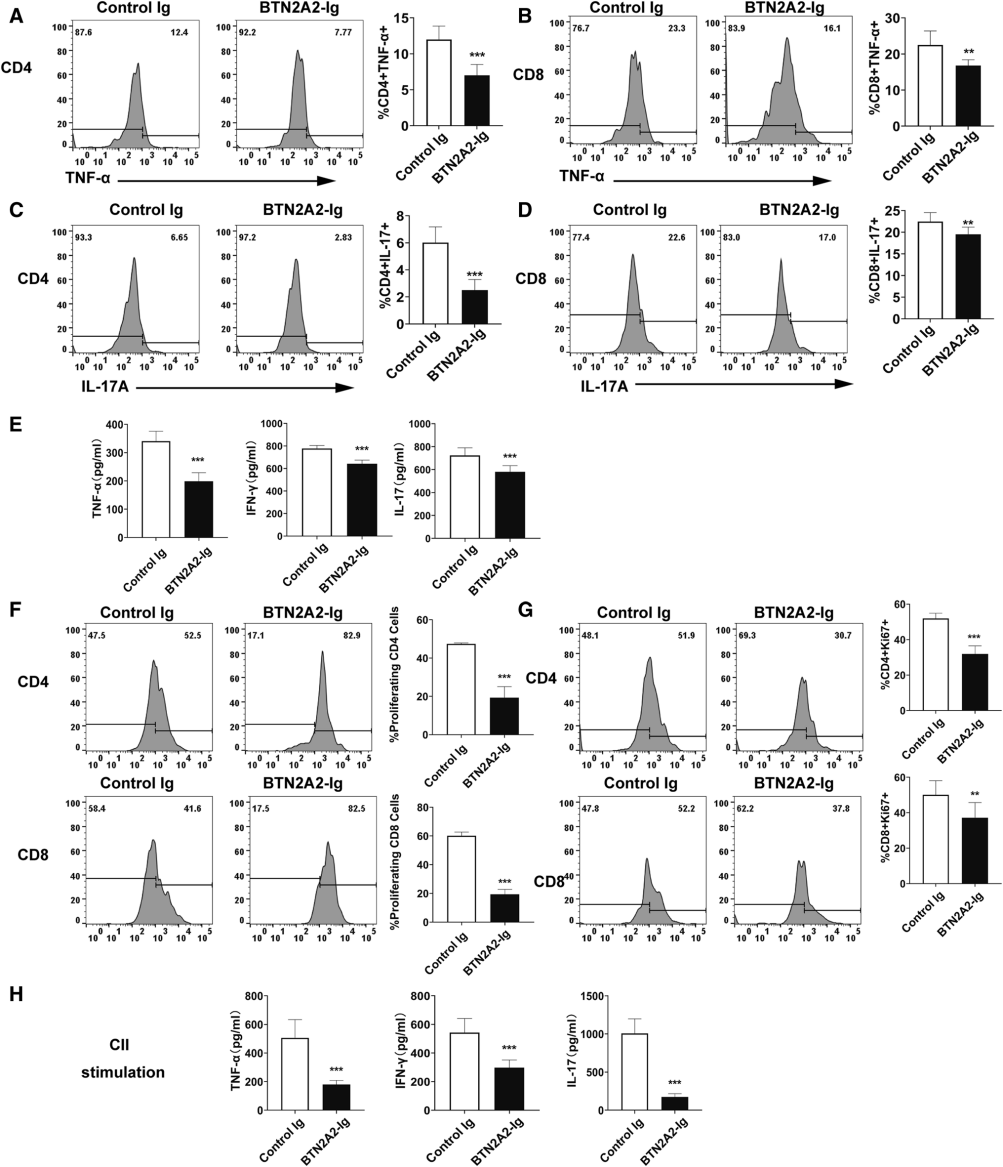
BTN2A2-Ig reduces Th1 and Th17 cytokine production in CIA mice. DBA/1J CIA mice were treated with mBTN2A2-Ig or control Ig as in Fig. 5. The percentages of cytokine—producing CD4 and CD8 T cells were analyzed by flow cytometry. The representative flow cytometric profiles and statistics analysis of (**A**,**B**) TNF-α^+^ or (**C**,**D**) IL-17A^+^ T cells. (**E**) The levels of TNF-α, IFN-γ, or IL-17A in the serum were measured by ELISA. (**F**–**H**) Splenocytes from CIA mice treated with BTN2A2-Ig or control Ig were cultured with 30 μg/ml of CII in vitro for 72 h. (**F**,**G**) T cell proliferation was analyzed by (**F**) CFSE dilution assay or (**G**) Ki67 staining. (**H**) The levels of IL-17A, TNF-α and IFN-γ in the supernatant were analyzed by ELISA. The data are expressed as the mean ± SD and representative of three independent experiments with similar results. (n = 6 each experiment each group) ***P < 0.001 compared with control Ig-treated mice.

We also analyzed cytokine production in the CIA mice. We first examined Th1 or Th17 cytokine-producing T cells in the spleen. As shown in (Fig. 6A–D), BTN2A2-Ig treatment led to reduced percentages of TNF-α and IL-17A-producing CD4 and CD8 T cells. We then examined the levels of Th1/Th17 cytokines in the serum. BTN2A2-Ig treatment also reduced the contents of TNF-α, IFN-γ and IL-17A in the serum (Fig. 6E).

We investigated whether BTN2A2-Ig modulates CII-specific T cell responses, the purpose of which is to detect antigen-specific T cell proliferation and cytokine production. Splenocytes from control Ig or BTN2A2-Ig-treated mice were isolated and stimulated with CII in vitro for 72 h. T cell proliferation was analyzed by CFSE dilution assay or Ki67 staining. As shown in (Fig. 6F,G), BTN2A2-Ig treatment led to reduced CD4 and CD8 T cell proliferation.

We then analyzed the cytokine contents in the supernatants of the CII-stimulated cultured cells. Splenic cells from the control Ig-treated mice produced high levels of IFN-γ and IL-17A in response to CII, whereas the production of IFN-γ and IL-17A was greatly reduced from the cells of BTN2A2-Ig-treated mice (Fig. 6H). Taken together, the results suggest that BTN2A2-Ig reduces CII-specific T cell proliferation and Th1 and Th17 cytokine production.

### BTN2A2-Ig reduces proinflammatory M1 macrophages but increases anti-inflammatory M2 macrophages in CIA mice

**Figure 7:**
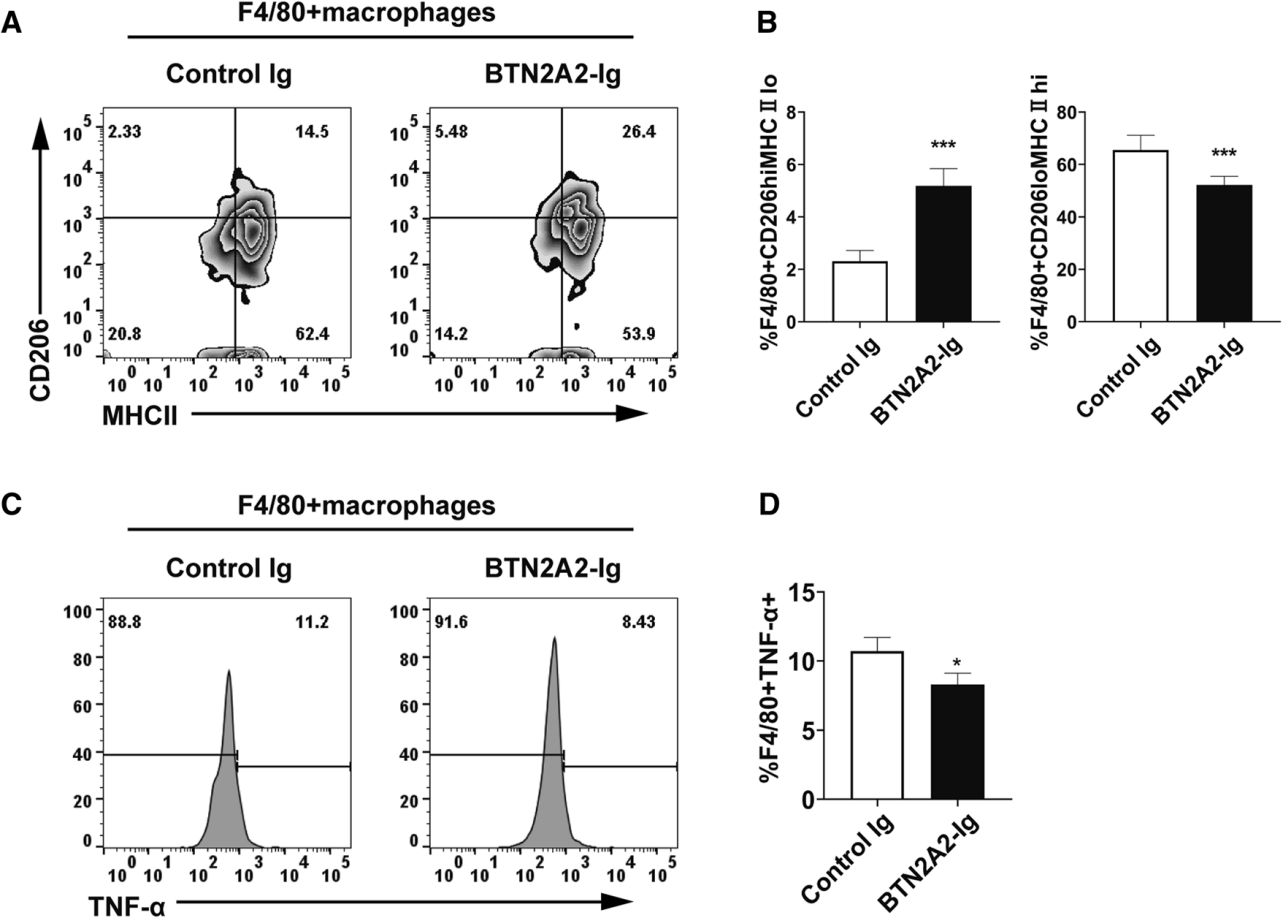
BTN2A2-Ig reduces M1 macrophages but increase M2 macrophages in vivo. Splenocytes from CIA mice treated with BTN2A2-Ig or control Ig as in Fig. 5. Cells were analyzed for (**A**,**B**) the percentages of F4/80^+^CD206^hi^MHCII^lo^ M2 and F4/80^+^CD206^lo^MHCII^hi^ M1, as well as (**C**,**D**) the percentages of TNF-α producing F4/80^+^ macrophages by flow cytometry. Data are shown as mean ± SD from one out of 3 independent experiments with similar results. (n = 6 each experiment each group) **P < 0.01, ***P < 0.001 compared with control Ig-treated mice.

Macrophages can be divided into two distinct subsets, the classically activated M1 and alternatively activated M2 macrophages^30^. M1 macrophages are proinflammatory, whereas M2 macrophages are anti-inflammatory. Since macrophages also express the BTN2A2 receptor, we analyzed macrophages in the BTN2A2-Ig-treated mice. BTN2A2-Ig reduced the percentage of CD206^lo^MHCII^hi^ M1 macrophages but increased the percentage of CD2O6^hi^MHCII^1o^ M2 macrophages (Fig. 7A,B). Furthermore, BTN2A2-Ig reduced the percentage of TNF-α producing F4/80^+^ macrophages (Fig. 7C,D). The results suggest that the CIA reduction by BTN2A2 may also be mediated by macrophages.

## Discussion

We have shown here that BTN2A2 is expressed on dendritic cells, macrophages and B cells, and its expression levels were significantly upregulated upon activation. The BTN2A2 putative receptor is expressed on activated CD4 and CD8 T cells at high levels and on activated dendritic cells, macrophages and B cells at low levels. BTN2A2-Ig protein inhibits the activation and proliferation of CD4 and CD8 T cells in vitro*.* Administration of BTN2A2-Ig protein ameliorates established CIA, which is related to reduced activation and proliferation of CD4 and CD8 T cells, decreased production of IFN-γ and IL-17A, and reduced inflammatory M1 macrophages but increased anti-inflammatory M2 macrophages. To our knowledge, this is the first time to show that in vivo administration of BTN2A2-Ig protein can attenuate autoimmune arthritis in mice.

T cell inhibitory molecules are usually expressed on APCs, which is involved in the regulation of T cell activation and proliferation. We show here that BTN2A2 is also expressed on APCs especially on activated DCs, macrophages and B cells, consistent with its role in limiting T cell activation and proliferation in inflammation. We have also shown that BTN2A2-Ig protein inhibits T cell activation as indicated by reduced expression of CD69 and increased proportion of naive T cells but decreased effector T cells after BTN2A2-Ig treatment. Furthermore, BTN2A2-Ig inhibits T cell proliferation. Our results are consistent with the data that the BTN2A2 putative receptor is expressed on activated T cells. The mechanisms by which BTN2A2 inhibits T cell functions are likely related to the inhibition of TCR signaling molecules including CD3s, Zap70, and Erkl/2, as well as PI3K and Akt signaling^19^.

BTN2A2-Ig treatment significantly reduces CIA clinical and pathological scores. T cell activation and proliferation in the BTN2A2-Ig-treated CIA mice were significantly reduced, which is agreement with our in vitro data. Furthermore, BTN2A2-Ig inhibits Th1/Th17 cytokine production of CII-specific autoreactive T cells. All these are likely responsible for the attenuated CIA by BTN2A2-Ig.

Tregs can inhibit effector T cells and are critical in immune tolerance induction^29^. We also found that CD4^+^CD25^+^FoxP3^+^ Tregs were increased in the BTN2A2-Ig-treated CIA mice. Tregs can be divided into natural and induced Tregs; the former develops in the thymus, whereas the latter develops in the periphery. Thymic epithelial cells (TECs) play a key role in natural Treg development. It has been reported that BTN2A2 is expressed on TECs^21^. It is possible that BTN2A2 is involved in the production of natural Tregs by its expression on TECs. Because BTN2A2 inhibits Erk1/2 and Akt signaling in T cells and because inhibition of these signaling increases the production of induced Tregs, it is possible that BTN2A2 also increases the production of induced Tregs.

BTN2A2-Ig-treated CIA mice also have reduced IL-17A producing T cells in the spleen and decreased IL-17A in the serum. It has been reported that Foxp3 can repress the expression of the IL-17 gene^31,32^. It is possible that the reduced Th17 cells in the BTN2A2-Ig-treated CIA mice is due to repression of IL-17 gene expression by Foxp3 or by induction of Tregs which suppress Th17 activation and proliferation^19^.

We have shown that the proportion of inflammatory M1 macrophages was decreased, whereas that of anti -inflammatory M2 macrophages was increased in BTN2A2-Ig-treated CIA mice, indicating that BTN2A2 also affects macrophages. It is likely that the effects of BTN2A2-Ig on macrophages also play a role in the ameliorated CIA. Although we have shown that the BTN2A2 putative receptor is expressed on macrophages, whether BTN2A2 directly affects macrophages remains to be investigated.

In summary, we have shown that BTN2A2-Ig protein inhibits T cell function in vitro and in vivo*.* Administration of BTN2A2-Ig protein attenuates established CIA. Our results suggest that BTN2A2-Ig protein has the potential to be used in the treatment of autoimmune disease including RA.

## Materials and methods

All methods were carried out in accordance with the relevant guidelines and regulations.

### Cloning and purification of mBTN2A2

The extracellular domain of mBTN2A2 (aa30-246) were cloned and insert into a pCMV6-AC-FC-S expression vector containing the constant region of mouse IgG2a (Origin Gene, Rockville, MD). The vector was transfected into HEK-293 cells. The fusion protein was purified from the supernatant using Protein G Sepharose 4 Fast Flow resin (GE Healthcare, Buckinghamshire, UK) according to the manufacturer's instructions. The purified protein was verified by SDS-PAGE, Coomassie staining, and western blotting. The protein was quantified using the Pierce BCA Protein Assay Kit (Pierce, Rockford, IL). The endotoxin level of the recombinant protein was < 0.01 EU/ml pig-1 of purified protein as determined by the endpoint chromogenic LAL test. Control Ig (recombinant mouse IgG2a Fc protein) was purchased from BXCell (West Lebanon, NH)^30^.

### SDS-PAGE and Western Blot

Purified mBTN2A2-Ig was loaded on a 12% SDS-PAGE, stained with Coomassie blue or transferred to a polyvinylidene fluoride membrane. The protein containing membrane was incubated with HRP conjugated anti-mouse IgG2 antibody, or anti-mBTN2A2 antibody (Novus Biologicals, Littleton, CO) followed by HRP conjugated second antibody, and then developed with Super Signal West Pico chemiluminescent Substrate (Thermo Fisher Scientific, Waltham, MA, USA)^33^.

### Mice

DBA/1 and C57BL/6 mice were purchased from the Gempharmatech Co. Ltd (Nanjing, China). The mice were maintained in specific pathogen-free rooms and used in accordance with protocols approved by the Institutional Animal Care and Use Committee of the Guizhou Medical University (NO:1900035). Furthermore, the study was carried out in compliance with the ARRIVE guidelines.

### Induction and assessment of CIA

Male 6–8 weeks DBA/1 mice were immunized with 200 pg of bovine collagen II (Chondrex Inc., cat. no. 2002-2) emulsified in an equal volume of Freund's complete adjuvant (CFA) at two injection sites of the base of the tails on day 0, and a boost injection of bovine collagen II emulsion in Freund's incomplete adjuvant (IFA) on 21 days. The mice were examined for signs of arthritis 2 times a week. When clinical scores were up to 8, the mice were injected i.p. with 15 pig BTN2A2-Ig or control Ig protein two times per week for 4 weeks. CIA development was monitored over time. The swelling of four paws was graded from 0 to 4 as follows^34^: grade 0, no evidence of erythema and swelling; grade 1, erythema and mild swelling confined to the tarsals or ankle joint; grade 2, erythema and mild swelling extending from the ankle to the tarsals; grade 3, erythema and moderate swelling extending from the ankle to metatarsal joints; and grade 4, erythema and severe swelling encompass the ankle, foot and digits, or ankylosis of the limb^2^. Each paw was graded, and the four scores were totaled so that the maximal scores per mouse was 16.

### Histopathology

Limbs were fixed in 4% paraformaldehyde and decalcified with 31.5% (v/v) formic acid and 13% (w/v) sodium citrate. The paraffin sections were stained with H&E. Subsequently, the evaluation of synovitis, pannus formation, as well as bone and cartilage destruction were determined by a graded scale as follows: grade 0, no signs of inflammation; grade 1, mild inflammation with hyperplasia of the synovial lining without cartilage destruction; grades 2 through 4, increasing degrees of inflammatory cell infiltrate and cartilage/bone destruction. Sections were also stained with 0.1% Safranin O for detection of cartilage proteoglycan and assessed by the Mankin scoring system: matrix staining (0–4), cartilage structure (0–6), cell appearance (0–3) and invasion of the tidemark (0–1)^35^.

### In vitro T cell assay

Splenocyte proliferative response was assessed by carboxyfluorescein diacetate succinimidyl ester (CFSE) dilution assay. Splenocytes were labeled with CFSE (Thermo Fisher Scientific, Waltham, MA, USA) and in the presence of BTN2A2-Ig or control Ig for 3 days. The cells were analyzed by flow cytometry.

### Flow cytometry analysis

Single-cell suspensions of spleen were stained with fluorochrome-conjugated Ab proteins as previously described. For intracellular staining, the cells were first permeabilized with 0.25% Triton X-100 for 20 min at 4 °C. Fluorochrome-conjugated antibodies were used for direct or indirect staining, including anti-CD4, CD8, CD19, CD11c, F4/80, CD44, CD62L, CD69, CD25, FoxP3, Ki67, CD206 and MHC II (BioLegend, San Diego, CA, USA). Anti-BTN2A2 polyclonal antibody and PE-conjugated secondary antibody were purchased from Novus Biologicals (Littleton, CO, USA) and Abcam (Cambridge, MA, USA). Isotype control antibodies were purchased from Abcam (Cambridge, MA, USA)^33^. BTN2A2-Ig was biotinylated with sulfo-NHS-LC-biotin (Thermo Fisher Scientific, Waltham, MA, USA). The samples were analyzed on a FACS Celesta (BD Biosciences). Data analysis was performed using FlowJo software (Ashland, OR, USA).

### Intracellular staining

For staining of intracellular TNF-αand IL-17A, splenocytes were stimulated by PMA (100 ng/ml), ionomycin (500 ng/ml) and brefeldin (100 ng/ml) (from Shanghai Selleck Chemicals) for 4 h at 37 °C, then stained with IL-17 and TNF-α antibody at 4 °C for 30 min, respectively; finally, the cells were stained with CD4 and CD8 antibody at 4 °C for 30 min. Cells were analyzed by flow cytometry^36^.

### Statistical analysis

All data are presented as the mean ± SD of at least three independent experiments. According to analysis, GraphPadPrim5 is used as a drawing tool and a secondary assist in statistical analysis tools. P-values were based on the two-sided Student’s t-test. P < 0.05 was considered to indicate a statistically significant difference.

## Supplementary Information

Supplementary Figures.

## Data Availability

The date that support the findings of this study are available from the corresponding author upon reasonable request.
