# Supplementary material for: BTN2A2 protein negatively regulates T cells to ameliorate collagen-induced arthritis in mice
Source: Sci Rep. 2021 Sep 29;11:19375. doi: 10.1038/s41598-021-98443-5 (PMC8481265; doi:10.1038/s41598-021-98443-5)
Supplement: Supplementary file 1 — Supplementary Figures. [file 41598_2021_98443_MOESM1_ESM.pdf]

Title： BTN2A2 protein negatively regulates T cells to ameliorate collagen-induced arthritis in mice

Authors: Xueping He[1], Rong Hu#[1,2], Peng Luo[3,4], Jie Gao[1], Wenjiang Yang[1], Jiaju Li[1], Youjiao Huang[1], Feng Han[5], Laijun Lai*[6], Min Su*[1,7,8]

Supplemental Figure 1.

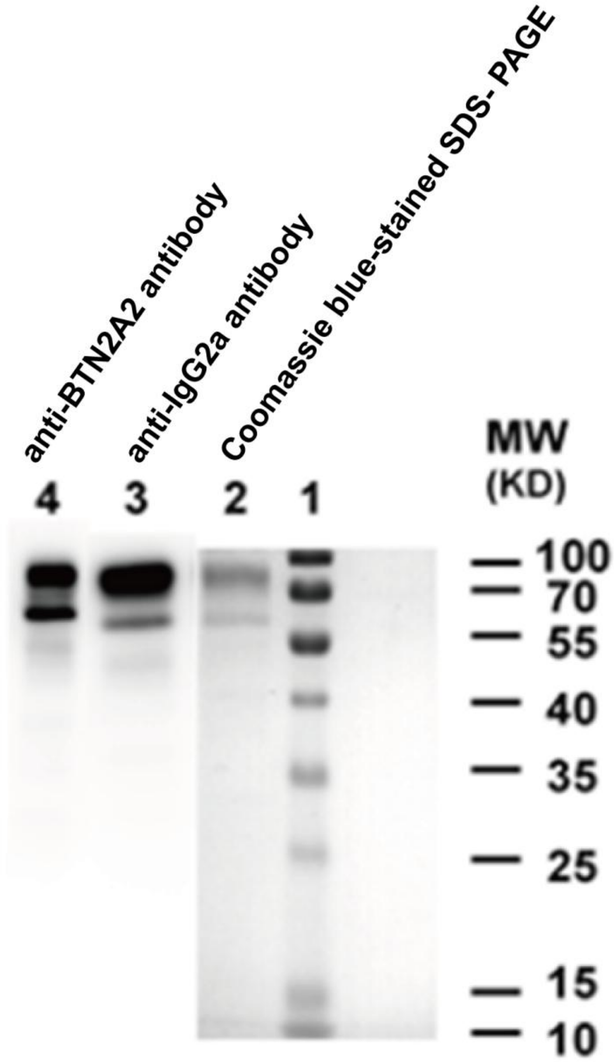

Supplemental Figure 1. Characterization of purified BTN2A2-Ig. Gel and blot show purified BTN2A2-Ig protein; Lane 1, MW markers; lane 2, Coomassie blue-stained SDS- PAGE; lane 3, Western blot with anti-IgG2a antibody; Lane 4, Western blot with anti-BTN2A2 antibody.

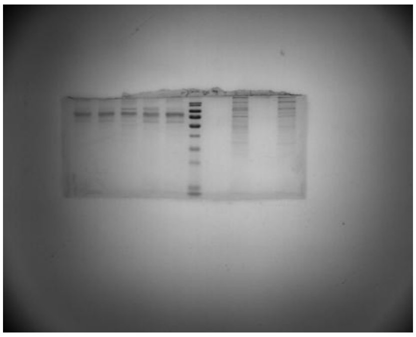

Lane 2: Coomassie blue stained SDS-PAGE original

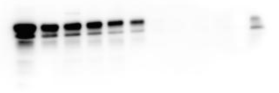

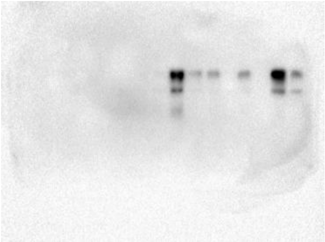

lane 3: Original image of western blot using anti-IgG2a.

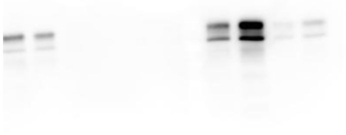

Lane 4： Original western blot of anti-BTN2A2 antibody.

Supplemental Figure 2.

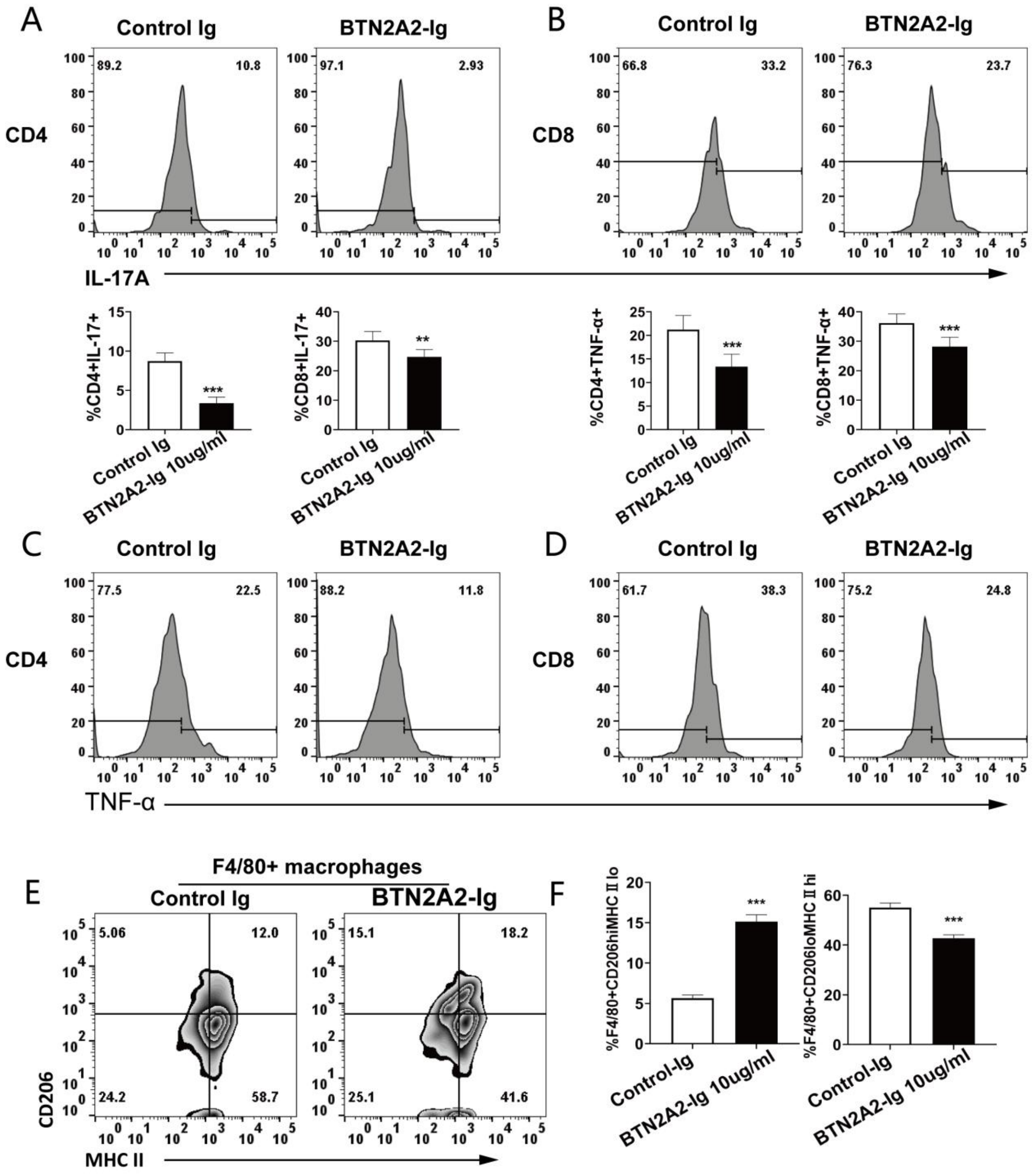

Supplemental Figure 2. Effects of BTN2A2-Ig on Th1 and Th17 cytokine-producing and the function and differentiation of macrophages in vitro. Spleen cells from C57BL/6 mice were incubated on a 96-well plate pre-coated with 1 μ g/ml anti-CD3 antibody and the specified dose of BTN2A2-Ig or control Ig(10 μg/ml). The percentages of cytokine - producing CD4 and CD8 T cells were analyzed by flow cytometry. The representative flow cytometric profiles and statistics analysis of (A, B) IL-17A$^+$ or (C, D) TNF-α$^+$ T cells. (E, F) the percentages of F4/80$^+$CD206hiMHCIIlo M2 and F4/80$^+$CD206loMHCIIhi M1. The data are representative of three independent experiments with similar results. (n=3 each experiment) **P＜0.01 and ***p < 0.001, compared with control Ig.
